# Digital-Assisted Community Pharmacy Cessation for Dual-Tobacco Users in Jordan: A Hybrid Cluster Randomized Controlled Trial

**DOI:** 10.3390/pharmacy14030077

**Published:** 2026-05-21

**Authors:** Derar H. Abdel-Qader, Nadia Al Mazrouei, Esra’ Taybeh, Rana Ibrahim, Abdullah Albassam, Eman Massad, Alia Saleh, Sahar Jaradat, Shorouq Al-Omoush

**Affiliations:** 1 Faculty of Pharmacy & Medical Sciences, University of Petra, Amman 11196, Jordan; aliasalehh95@gmail.com (A.S.); sahar.jar279@icloud.com (S.J.); shoroqalomoush@gmail.com (S.A.-O.); 2 Department of Pharmacy Practice and Pharmacotherapeutics, College of Pharmacy, University of Sharjah, Sharjah 27272, United Arab Emirates; nalmazrouei@sharjah.ac.ae (N.A.M.); ribrahim@sharjah.ac.ae (R.I.); 3 Department of Applied Pharmaceutical Sciences, School of Pharmacy, Isra University, Amman 11622, Jordan; esra.taybeh@iu.edu.jo; 4 Department of Pharmacy Practice, Faculty of Pharmacy, Kuwait University, Safat 12037, Kuwait; albassam@hsc.edu.kw; 5 Public Health Institute, The University of Jordan, Amman 11196, Jordan; e.massad@ju.edu.jo

**Keywords:** smoking cessation, community pharmacy services, dual-tobacco use, waterpipe smoking, digital health, implementation science, cluster randomized controlled trial

## Abstract

Tobacco use remains a major public health challenge in Jordan, where cigarette smoking and waterpipe use are both common and dual use is increasingly prevalent. Community pharmacies are highly accessible healthcare settings, yet structured smoking-cessation services remain underutilized. This study evaluated the clinical effectiveness and implementation of Dual-Quit Digital, a pharmacist-delivered cessation counseling program tailored to the type of tobacco used, paired with a 6-month automated WhatsApp^®^ (Menlo Park, CA, USA) follow-up system. We conducted a pragmatic, two-arm, parallel-group, Hybrid Type 2 cluster randomized controlled trial in 16 community pharmacies in Jordan, randomized 1:1 to intervention or usual care. A total of 320 adult tobacco users were enrolled (160 per arm). The intervention combined a structured in-pharmacy pharmacist consultation, tailored behavioral support, phenotype-stratified pharmacotherapy support, and 6 months of semi-automated WhatsApp^®^ follow-up with telepharmacy escalation for predefined red-flag responses. The control arm received usual care, consisting of opportunistic brief advice and standard over-the-counter sales without proactive follow-up. The primary outcome was biochemically verified continuous abstinence at 6 months, defined as exhaled carbon monoxide (CO) < 10 ppm and analyzed using intention-to-treat principles. Secondary outcomes included 7-day point prevalence abstinence (PPA) at 3 and 6 months, 30-day PPA at 6 months, both-product abstinence among baseline dual users, pharmacotherapy uptake and adherence, and implementation-relevant outcomes, including service reach, feasibility of recruitment, and digital engagement metrics. All 16 pharmacies were retained, and all 320 randomized participants were included in the intention-to-treat analysis. At 6 months, CO-verified continuous abstinence was achieved by 26.3% of participants in the intervention arm compared with 11.3% in the control arm (adjusted odds ratio [aOR] 2.84, 95% CI 1.55–5.18; *p* < 0.001). The intervention also improved 7-day PPA at 3 months (33.1% vs. 15.6%; aOR 2.68, 95% CI 1.56–4.60; *p* < 0.001), 7-day PPA at 6 months (30.6% vs. 14.4%; aOR 2.62, 95% CI 1.48–4.62; *p* = 0.001), and 30-day PPA at 6 months (28.1% vs. 11.9%; aOR 2.89, 95% CI 1.59–5.24; *p* < 0.001). Among baseline dual users, both-product abstinence was higher in the intervention arm (21.9% vs. 7.8%; aOR 3.30, 95% CI 1.12–9.75; *p* = 0.026). Pharmacotherapy initiation was more frequent in the intervention arm (72.5% vs. 28.1%; *p* < 0.001), as was self-reported adherence for at least 8 weeks among initiators (56.0% vs. 26.7%; *p* = 0.002). In the intervention arm, active patient response rates to scheduled WhatsApp^®^ messages remained substantial, with 88.1% responding at Week 1, 73.8% at Week 4, 67.5% at Month 3, and 61.3% at Month 6; 145 red-flag triggers were captured from 62 participants, and 84.1% of escalations resulted in successful pharmacist follow-up within 48 h. The Dual-Quit Digital model significantly improved smoking-cessation outcomes compared with usual care and proved operationally feasible. These findings support integrating phenotype-stratified pharmacist counselling, pharmacotherapy support, and low-burden digital follow-up as a pragmatic cessation model for Jordan and similar settings.

## 1. Introduction

Tobacco use remains a major public health challenge in Jordan. Official reports reveal that the prevalence of smoking is exceptionally high, especially among men, exceeding 65% [1]. In addition, the use of multiple tobacco products, including manufactured cigarettes, waterpipe, heated tobacco, and electronic cigarettes, is significant. This pattern of multi-product tobacco use has also been reflected in Jordanian research; in a national survey, Abdel-Qader and Al Meslamani [2] reported that current e-cigarette use among adults in Jordan was 11.7% and documented concurrent dual smoking among 4.0% of respondents, alongside widespread misconceptions, including beliefs that vaping is not harmful to children and pregnant women (73.1%), is not addictive (58.2%), and can help with smoking cessation (69.1%). In 2024, the Kingdom of Jordan announced a national strategy to combat tobacco and smoking from 2024 to 2030, reflecting the issue of tobacco control and smoking cessation is a priority public health issue in the country [1]. The expansion of access to effective cessation treatments is a key priority. Current evidence and international recommendations all point to the effectiveness of both behavioral counselling and pharmacotherapy, with the best outcomes seen when the two approaches are used together [3,4]. Community pharmacies have the potential to play a part in this process. They are highly accessible and frequently visited, and their role is supported by evidence from the Cochrane review that found more intensive smoking cessation interventions delivered by community pharmacy staff were more effective compared with less intensive care for smoking cessation [5]. Yet the translation of this potential into action in the context of Jordan is currently limited. A study among community pharmacists in Northern Jordan found that while attitudes towards smoking cessation were positive, actual cessation practice levels specifically within the community pharmacy setting were poor, with significant barriers to action including the lack of training on nicotine replacement therapy (NRT), the lack of smoking cessation programs, and the lack of demand from smokers themselves [6].

The second challenge is that much of the literature on tobacco cessation has traditionally emphasized exclusive cigarette smokers, but tobacco use in Jordan and the Eastern Mediterranean Region more generally includes waterpipe or multiple tobacco products. This is important because of the social, cue, and perception factors associated with waterpipe use, as well as the complex factors related to dual or multiple tobacco use, which could impact factors related to nicotine dependence, relapse, and treatment planning. A recent Cochrane review of the literature emphasized the importance of behavioral interventions to increase the rate of quitters for waterpipe smokers, but noted the limited evidence for pharmacological interventions and the lack of studies of dual or multiple tobacco use, which requires more targeted studies [7]. With regard to service delivery, there are also challenges. In the STOP cluster randomized trial, for example, a positive effect of staff training on perceived skill was found, but there was no significant effect on throughput, retention, or quit rates, highlighting the importance of a practical system to support these efforts [8].

Digital follow-up might help bridge this implementation gap. The evidence from the Cochrane review conducted by Whittaker et al. [9] suggests that computer-tailored text messaging interventions increase quit rates in comparison to minimal support and also increase quit rates when added to other interventions to aid in smoking cessation. However, there is a scarcity of evidence to support stand-alone interventions using smartphones. This might imply that a more familiar messaging system might be a more viable option than a stand-alone app [9]. In Jordan, where smartphone penetration exceeds 90% in 2024 [10] and WhatsApp^®^ (Menlo Park, CA, USA) is the dominant messaging platform across almost all adult age groups and socioeconomic demographics, and pharmacist time is often constrained, a semi-automated messaging system might be a viable solution to increase adherence support.

Against this background, we conducted a pragmatic Hybrid Type 2 cluster randomized controlled trial to evaluate the clinical effectiveness and implementation of Dual-Quit Digital, a structured, tobacco-use phenotype-stratified community-pharmacy smoking-cessation service for adult tobacco users in Jordan. The intervention combined phenotype-stratified pharmacist counselling and pharmacotherapy support with a semi-automated WhatsApp^®^ follow-up and relapse-prevention pathway. We hypothesized that, compared with usual care, this model would improve biochemically verified abstinence at 6 months while also demonstrating operational feasibility within routine community-pharmacy practice.

## 2. Methods

### 2.1. Study Design

This study was a pragmatic, two-arm, parallel-group, cluster randomized controlled trial (cRCT) using a Hybrid Type 2 effectiveness–implementation framework. Community pharmacies are the unit of randomization (clusters) to minimize contamination across staff within the same pharmacy, consistent with guidance for cluster trials. The design and reporting followed CONSORT 2010 guidance for individually randomized, parallel-group trials [11].

Hybrid Type 2 effectiveness-implementation framework was selected to accelerate the translation of research into practice. Curran et al. [12] defined it by stating “We need to prove the clinical intervention works (effectiveness) AND we need to prove the delivery method works (implementation) at the same time”. Given the urgent public health burden of tobacco in Jordan, this design allows for the simultaneous evaluation of the clinical effectiveness of the Dual-Quit Digital intervention (measured by biochemically verified abstinence) and implementation-relevant outcomes focused on service uptake and operational feasibility (including cluster participation and retention, recruitment feasibility, participant engagement with the WhatsApp^®^ follow-up pathway, telepharmacy escalation performance, and pharmacotherapy uptake and adherence). This approach was intended to determine not only whether the intervention improved smoking-cessation outcomes, but also whether it could be feasibly delivered and sustained within real-world pharmacy workflows in Jordan.

**Figure 1:**
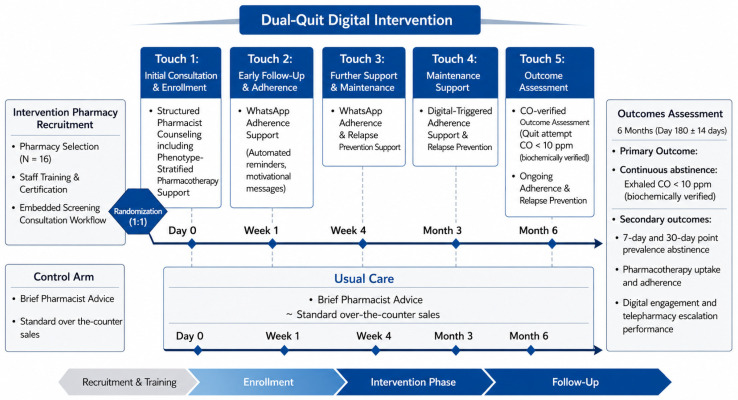
Schematic representation of the study design and timeline.

A schematic overview of the trial design, participant flow, and intervention timeline is presented in Figure 1.

The flow diagram outlines the cluster-randomized trial comparing the Dual-Quit Digital intervention to Usual Care. The intervention arm features a pharmacist-led baseline consultation (Day 0) followed by a 6-month digital adherence and relapse-prevention pathway via WhatsApp^®^, comprising five core touchpoints (Day 0, Week 1, Week 4, Month 3, and Month 6). The control arm consists of standard over-the-counter care and opportunistic brief advice without proactive follow-up. Both arms underwent identical assessment schedules, with the primary clinical outcome being biochemically verified (exhaled CO < 10 ppm) continuous abstinence evaluated at 6 months. Continuous abstinence was defined as self-reported abstinence from all combustible tobacco from the target quit date to the follow-up, consistent with the Russell Standard approach to cessation outcomes [13]. Target quit date was defined as a specific, predefined calendar date on which a participant formally commits to initiating complete abstinence from all targeted tobacco products [13]. For this study, a 30-day window was considered for all participants.

### 2.2. Ethics and Participants Consent

Ethical approval was obtained from the Institutional Review Board (IRB) at University of Petra (S/17/4/2025). Written consent was taken from all individual participants.

### 2.3. Setting, Participants, and Sample Size

This cRCT was implemented in community pharmacies across Jordan, using a stratified, nationally representative sampling approach. Administratively, Jordan’s 12 governorates are grouped into three regions; North (Mafraq, Irbid, Ajloun, Jerash), Central (Amman, Zarqa, Balqa, Madaba), and South (Ma’an, Aqaba, Karak, Tafilah). The sampling frame was derived from the official registry maintained by Jordan Pharmacists Association (JPA). Based on JPA (2025) records, Jordan has approximately 5048 licensed community pharmacies [14]. For this study, the registry extract was subdivided into three non-overlapping geographic strata: Central Region (70.4%), North Region (23.9%), and South Region (5.6%). Within each stratum, pharmacies were selected by computer-generated simple random sampling. To preserve national representativeness with a fixed total of 16 (8 pairs) clusters, proportional allocation was used (rounded to whole pharmacies), yielding 10 (5 pairs) Central, 4 (2 pairs) North, and 1 (1 pair) South pharmacy.

Participating pharmacies (clusters) were eligible if they are licensed community pharmacies in Jordan, employ at least one full-time pharmacist and at least one pharmacy assistant, and have a private or semi-private space suitable for brief counselling and Carbon Monoxide (CO) verification procedures. Pharmacies were willing to complete the study’s one-day training workshop and implement the protocolized workflow (counter-staff screening/referral, pharmacist consultation, and intervention documentation). Pharmacies were excluded if they are hospital-based or if they are currently participating in other smoking cessation research that could contaminate delivery or outcomes.

Individual participants were eligible if they were adults (≥18 years), currently use tobacco (cigarettes, waterpipe/shisha, or dual-use), intend to make a quit attempt within the next 30 days, have a smartphone with active WhatsApp^®^ access, and can provide written informed consent. Individuals were excluded if they were already enrolled in another structured smoking cessation program, if they have contraindications to NRT or varenicline (e.g., severe cardiovascular events, pregnancy/breastfeeding, history of seizures or specific psychiatric conditions, and hypersensitivity), in which case they were referred to appropriate specialist care. Because of the cluster-randomized design, patient participants were only informed about the cessation support available at their specific pharmacy during the consent process; they were unaware of the trial’s overall comparative design or the existence of an alternate arm. No financial incentives were provided to patient participants in order to accurately reflect real-world engagement, though participating pharmacies received a nominal stipend to cover the administrative time required for study documentation.

Pharmacy recruitment, site initiation, and cluster randomization were conducted during June 2025. Following cluster randomization, individual participant recruitment commenced. To ensure the study achieved adequate statistical power, individual recruitment remained open continuously across all participating sites until the predefined target sample size of 320 participants was reached spanning from July 2025 to August 2025. The 6-month follow-up period concluded in February 2026.

The sample size was calculated to detect a clinically meaningful difference in biochemically verified continuous abstinence at 6 months for a pharmacist-led cessation service with a messaging-based digital follow-up (WhatsApp^®^) delivered through community pharmacies in Jordan. Effect-size assumptions were informed by high-quality synthesis evidence showing that structured community-pharmacy cessation interventions increase quitting versus usual care (risk ratio [RR] ≈ 2.30) [5] and that mobile text-messaging support improves cessation outcomes (RR ≈ 1.59) [9].

Conservatively, we assumed a 6-month continuous abstinence rate of 10% in the control arm, consistent with usual care rates in the Middle East and general population estimates [15,16]. For the intervention arm, we anticipated a rate of 26% (absolute difference ~16%). This is aligned with a recent pragmatic trial of structured pharmacist-led programs, which has demonstrated abstinence rates ranging from 22% to 36% [16]. Under individual randomization, this requires ~90 participants per arm (80% power; two-sided α = 0.05).

Because this is a cluster randomized trial (pharmacy as the unit of randomization), we inflated the sample using the standard design effect, DE = 1 + (m − 1) × ICC [17], assuming mean cluster size m = 20 patient participants per pharmacy and intra-cluster correlation coefficient ICC = 0.015 [18]. This yields DE = 1.285 and an adjusted requirement of ~116 participants per arm; allowing 25% loss to follow-up increases the target to ~145 per arm (290 participants).

We divided the total required sample size (290 participants) by the expected cluster size (20 participants per pharmacy), yielding 14.5 total pharmacies. However, to accommodate the cluster-randomized design which requires an equal number of clusters per arm, we rounded the number of required pharmacies up from 14.5 to 16 (8 per arm). This results in a final total sample size of 320 participants. This slight over-recruitment provides a safety buffer against potential variability in cluster sizes.

### 2.4. Interventions

#### 2.4.1. Control Group (Usual Care)

Participants in the control group received routine community-pharmacy practice. This unstructured standard of care was consistent across all control pharmacies and was strictly limited to opportunistic smoking-cessation advice only when requested by the patient or when initiated by staff, and standard over-the-counter supply of cessation aids (e.g., NRT) according to usual dispensing practice. Study personnel verified that no proactive follow-ups or digital outreach were initiated by control pharmacies during the study period. Control pharmacies did not implement a structured phenotype-stratified cessation workflow, did not schedule proactive follow-up contacts, and did not provide a WhatsApp^®^-based adherence or relapse-prevention pathway. Patient participants were contacted only at the pre-specified follow-up timepoints to collect outcome data, and no additional counselling, clinical troubleshooting, or digital relapse-prevention prompts were provided between these assessments.

#### 2.4.2. Intervention Group: The “Dual-Quit Digital” Community-Pharmacy Program

Participants in the intervention group received a structured cessation support program delivered through a whole-of-pharmacy workflow, combining (i) standardized in-pharmacy behavioral and pharmacotherapy support and (ii) a semi-automated WhatsApp^®^ follow-up layer for 6 months. The program was informed by evidence that structured pharmacy personnel interventions improved cessation outcomes compared with less intensive care, and that mobile messaging interventions increased quit rates when used as adjunct support [5,9].

Pharmacy assistants used brief scripts and visible prompts to identify current tobacco users (defined as any self-reported use of combustible tobacco (cigarettes and/or waterpipe/shisha) in the period immediately preceding enrollment, as captured on the baseline intake form) and referred eligible and interested individuals to the on-site pharmacist for enrollment and consultation.

- Touch 1—Pharmacist consultation (Day 0 ± 2 days; baseline visit): The pharmacist delivered a structured 10–15 min consultation that assessed tobacco-use phenotype (exclusive cigarette, exclusive waterpipe, or dual use), dependence/severity (measured using the Fagerström Test for Nicotine Dependence [FTND]), prior quit attempts and readiness to quit. Baseline tobacco-use phenotype was defined as each participant’s combustible-tobacco use pattern in the 30 days preceding enrollment, and it was classified into three mutually exclusive categories using structured self-report items adapted from standardized tobacco surveillance questions [19]. Participants were categorized as follows [20]: (1): exclusive cigarette users reported current combustible cigarette smoking (operational threshold: ≥1 cigarette/day on average) and reported no waterpipe sessions, (2): exclusive waterpipe/shisha users reported current waterpipe use (operational threshold: ≥1 session/week on average, reflecting the typical episodic but highly concentrated nicotine exposure characteristic of waterpipe smoking) and reported no combustible cigarette smoking and (3) dual users met both operational thresholds (cigarettes and waterpipe). A target quit date within 30 days was agreed upon, and participants received a tailored quit plan that combined behavioral strategies (trigger management, coping planning, lapse management) with phenotype-stratified pharmacotherapy support. Pharmacotherapy international guidance [4] followed clinical recommendations that behavioral support combined with effective cessation medicines improved outcomes. Phenotype-stratified pharmacotherapy support was delivered by a pharmacist using a protocolized decision algorithm. The pharmacist documented the phenotype classification, completed a brief safety screen for contraindications and precautions, and then recommended and supported initiation of evidence-based cessation pharmacotherapy (e.g., NRT and, where clinically appropriate through routine care pathways, prescription options such as varenicline/bupropion), consistent with World Health Organization clinical treatment guidance [4]. Phenotype-stratified pharmacotherapy support was delivered by a pharmacist using a protocolized decision algorithm. Treatment varied according to the participant’s baseline tobacco-use phenotype [21]: (1) exclusive cigarette users (were primarily directed toward standard NRT or varenicline; (2) xclusive waterpipe/shisha users focused heavily on behavioral cue management and (3) dual users were recommended combination NRT (e.g., long-acting patch plus short-acting gum) to combat complex, overlapping withdrawal profiles. For dual users, the clinical protocol designated combination NRT (e.g., a long-acting patch plus short-acting gum) to address both baseline withdrawal and cue-driven cravings as the first-line, preferred recommendation [22]. Where appropriate and feasible, participants were supported to initiate evidence-based cessation pharmacotherapy (NRT, varenicline, or bupropion). Where appropriate, meaning in the absence of clinical contraindications and within the local scope of practice or collaborative practice agreements, pharmacists directly initiated or managed prescription cessation medications (e.g., varenicline, bupropion), thereby maximizing access while continuing behavioral and adherence support (which included dosage troubleshooting and managing minor side effects).
- Touch 2—Early digital support (Week 1 ± 2 days): Participants received WhatsApp^®^ check-ins focused on quit-day preparation or early abstinence support (depending on quit date within 30 days), including quick-reply prompts to assess cravings, lapses, medication use, and withdrawal symptoms. Automated messages provided brief coping strategies and reminders aligned with the participant’s quit plan.
- Touch 3—Consolidation support (Week 4 ± 2 days): Participants received relapse-prevention prompts and a structured WhatsApp^®^ check-in assessing adherence to pharmacotherapy, breakthrough cravings, and high-risk situations (including waterpipe-related social exposures). Participants reporting a lapse or high-risk responses were flagged for pharmacist follow-up.
- Touch 4—Maintenance support (3 Month ± 2 days ± window): Participants received a scheduled digital maintenance check-in targeting longer-term relapse prevention, problem-solving for recurrent triggers, and reinforcement of coping plans.
- Touch 5—Long-term maintenance and relapse prevention (Month 6 ± 2 days): Participants received a final scheduled WhatsApp^®^ maintenance check-in and were guided through relapse-prevention planning for sustained abstinence and/or step-up support if they had lapsed.

Structured prompts were delivered via an automated WhatsApp^®^ Business API managed by the central study team. When patient participants reported predefined red-flag responses (e.g., lapse, severe cravings, medication intolerance, or inability to adhere), the automated system autonomously flagged the keywords and sent an immediate notification to the specific dispensing pharmacist. The pharmacist was then solely responsible for executing a targeted telepharmacy follow-up phone/WhatsApp^®^ call to provide counselling, adjust the quit plan, and reinforce appropriate medication use within scope and local practice pathways. The digital follow-ups were automatically scheduled by the software system relative to the patient’s chosen target quit date (Day 0).

To ensure intervention fidelity and consistent delivery across sites, pharmacy staff involved in the intervention completed a standardized one-day training workshop covering the workflow, scripts, documentation tools, phenotype stratification, and safety screening. Standardized consultation guides and message templates were used to reduce inter-provider variability.

WhatsApp^®^ was used to deliver structured prompts, collect brief responses, and support scheduling and escalation. Clinical interventions and outcome data were recorded in the study database rather than relying on chat logs as the primary clinical record, and participation in WhatsApp^®^ follow-up was contingent on explicit participant consent.

### 2.5. Outcomes

#### 2.5.1. Primary Clinical Effectiveness Outcome

Continuous abstinence at 6 months was biochemically validated at the 6-month assessment using a portable exhaled CO breath monitor (CO Check Pro, MD Diagnostics Ltd., Amman, Jordan), with a threshold of CO < 10 ppm [21]. A threshold of CO < 10 ppm was chosen over <7 ppm due to the high prevalence of concurrent waterpipe use and the uniquely high environmental/second-hand smoke exposure common in Jordan, aligning with validated regional guidelines [21]. Exhaled CO readings captured by the device are classified into four clinical thresholds: non-smoker (Green: 0–6 ppm), light smoker (Yellow: 7–10 ppm), smoker (Red: 11–20 ppm), and heavy smoker (Flashing Red: >20 ppm). Cotinine/saliva tests were not used as the primary biochemical validator because participants could have used NRT or non-combustible nicotine products as part of their quit attempt, which limited cotinine’s specificity for combustible smoking.

#### 2.5.2. Secondary Clinical Effectiveness Outcomes

**7-day and 30-day PPA:** The 7-day PPA outcome was assessed at both 3 and 6 months, while 30-day point prevalence abstinence was assessed at 6 months. It was validated using exhaled CO at the same threshold used for the primary outcome [21]. Point prevalence abstinence (PPA) was defined as self-reported no combustible tobacco use during the specified recall period preceding each follow-up assessment.

**Dual-user both-product cessation (baseline dual users only):** Among participants classified as dual users at baseline, the proportion who achieved abstinence from both cigarettes and waterpipe/shisha at 6 months was measured, alongside the proportion who quit only one product (partial cessation). E-cigarette users were excluded because the primary biochemical validator (CO monitor) does not detect non-combustible nicotine use.

**Pharmacotherapy utilization and adherence (where initiated by the patient participant):** Use of cessation pharmacotherapy and adherence patterns were recorded during follow-ups and through routine service documentation, consistent with guideline emphasis on combining behavioral support with effective cessation pharmacotherapy [4].

**Digital engagement (intervention arm:** Engagement with the digital follow-up layers (Weeks 1 & 4 and Months 3 & 6) was quantified as the percentage of scheduled WhatsApp^®^ check-ins responded to and the number of automated red-flag triggers (e.g., a patient texting that they had a lapse or severe cravings) that resulted in pharmacist telepharmacy escalation. The automated system generated a digital alert that was sent via a secure portal directly to the pharmacist on duty.

**Telepharmacy escalation performance**: The number of automated red-flag triggers captured during follow-up and the proportion of escalations resulting in successful pharmacist follow-up within 48 h.

**Pharmacotherapy implementation**: the initiation of any evidence-based cessation pharmacotherapy, use of NRT among dual-users and self-reported continuous pharmacotherapy use for at least 8 weeks among those who initiated treatment.

#### 2.5.3. Implementation-Relevant Outcomes

In keeping with the hybrid effectiveness–implementation design, the operational feasibility of delivering the Dual-Quit Digital model in community pharmacies was assessed using both cluster-level metrics and the secondary engagement metrics defined above (specifically, WhatsApp response rates and telepharmacy escalation completion). These outcomes included:

- **Cluster participation/retention**: The proportion of randomized intervention pharmacies that remained active throughout 6 months follow-up.
- **Recruitment feasibility**: The achievement of the planned participant enrollment target within the predefined recruitment period.
- **Pharmacotherapy implementation**: Evaluated using the utilization and adherence metrics defined above (Section 2.5.2) to measure the extent to which pharmacists successfully adopted the phenotype-stratified algorithm and integrated evidence-based treatment recommendations into their routine workflow.
- **Service Adoption and Fidelity**: Evaluated using the digital engagement, and telepharmacy escalation performance metrics (defined in Section 2.5.2) to determine if the clinical workflow could be sustainably delivered within real-world pharmacy operations.

### 2.6. Randomization, Allocation Concealment and Blinding

After pharmacy recruitment and baseline site characterization, pharmacies were randomized 1:1 by an independent statistician using a computer-generated sequence. To improve balance, randomization was stratified by key pharmacy characteristics (governorate). Allocation was concealed from recruiting pharmacies until assignment, and the sequence was stored by the statistician.

Blinding of pharmacists and participants is not feasible due to the behavioral and workflow nature of the intervention. However, patient participants were effectively blinded to the comparative design during the consent process, minimizing expectation bias. Moreover, clinical outcomes were assessed by trained research assistants from the study team (not the treating pharmacists) who were blinded to allocation. Biochemical verification was conducted in person within the private consultation spaces of the respective pharmacies.

### 2.7. Data Sources and Data Collection Schedule

Participant-level data were collected via structured electronic case report forms. Baseline data were collected by the pharmacist via structured electronic tablet forms during the initial consultation. This included demographic information, tobacco use phenotype, dependence indicators, quit history, comorbid conditions, and exhaled CO levels. Follow-up data were collected at pre-specified time points based on the study pathway, which included Week 1, Week 4, Month 3, and Month 6.

Clinical effectiveness data were collected on self-reported abstinence, biochemical verification of abstinence at 6 months, and the use/adherence to pharmacotherapy. Data relevant to implementation were collected from the screening logs, pharmacy participation logs, intervention documentation forms, and the digital follow-up process. Data on digital engagement were collected from the scheduled WhatsApp^®^ contacts, while data on telepharmacy escalations were collected from the pre-specified red flags identified from the intervention process and the corresponding follow-up process with the pharmacist. Cluster participation and retention were recorded from the pharmacy recruitment and completion records. Feasibility of recruitment was assessed based on the proportion of the target sample size that was recruited over the planned period. WhatsApp^®^ was used as a channel for communicating with the participants. However, the clinical and research data were entered into the database rather than the chat logs (Supplementary Material).

During June 2025, a total of 24 community pharmacies across Jordan were assessed for eligibility. Of these, 5 pharmacies did not meet the inclusion criteria, and 3 declined to participate due to time constraints. The remaining 16 pharmacies were recruited and randomized 1:1, with 8 pharmacies assigned to the Dual-Quit Digital intervention arm and 8 pharmacies assigned to the usual care (control) arm.

Individual participant recruitment took place between July 2025 and August 2025. Within the 16 clusters, a total of 415 adult tobacco users were screened by pharmacy staff. Of these, 95 were excluded (42 did not meet inclusion criteria, 38 were not ready to set a quit date within 30 days, and 15 declined to participate). The final sample of 320 participants provided written informed consent and were enrolled (160 in the intervention group and 160 in the control group). The final 6-month follow-up assessments were completed in February 2026.

At the 6-month primary endpoint, individual participant retention was 78.1% in the intervention group (125/160) and 67.5% in the control group (108/160). As per the Intention-To-Treat (ITT) protocol, all 320 randomized participants were included in the primary analysis, with missing data imputed as smoking. No pharmacies withdrew from the study; therefore, the cluster-level retention was 100%.

### 2.8. Statistical Analysis

The analysis was conducted using SPSS Statistics version 28. The analysis was in accordance with the ITT principles, with missing primary outcome values imputed using the missing = smoking convention. For the primary outcome, a mixed effects logistic regression was utilized, with treatment arm as a fixed effect and pharmacy (cluster) as a random effect.

In addition to the primary mixed effects logistic regression analysis of the primary outcome, secondary and implementation-relevant outcomes were analyzed using appropriate techniques based on the measurement level of the outcome. For categorical outcomes, the results are provided as frequency and percentage, while for continuous outcomes, the results are provided as mean and standard deviations. For pharmacotherapy-related outcomes, between-group comparisons were carried out using cluster-adjusted chi-square tests for categorical variables and mixed-effects linear regression for continuous variables in accordance with the overall analytic framework of the trial. For implementation-relevant outcomes that are specific to the intervention arm, such as digital engagement and telepharmacy escalation metrics, the analysis was carried out using descriptive techniques.

## 3. Results

### 3.1. Baseline Characteristics of Participants

Table 1 shows that the intervention and control groups were well balanced at baseline across demographic, tobacco-use, clinical, and motivational variables, with no statistically significant between-group differences observed for any measured characteristic (all *p* > 0.05). Participants were predominantly male in both groups (intervention: n = 148, 92.5%; control: n = 151, 94.4%), and the largest age category was 30–39 years (intervention: n = 62, 38.8%; control: n = 58, 36.3%). Educational attainment and marital status were similarly distributed between study arms. Tobacco-use phenotype was also comparable, with similar proportions of exclusive cigarette smokers (45.0% vs. 43.8%), exclusive waterpipe/shisha users (15.0% vs. 16.3%), and dual users (40.0% vs. 40.0%) in both groups.

Baseline smoking-related indicators were likewise similar between arms. Most participants had used tobacco for more than 10 years (intervention: n = 26, 16.3%; control: n = 23, 14.4%), while the mean number of cigarettes smoked per day among cigarette/dual users and the mean number of waterpipe sessions per week among waterpipe/dual users did not differ significantly between groups (*p* = 0.495 vs. *p* = 0.380, respectively). Baseline FTND and exhaled CO levels were also comparable. In addition, prior quit attempts, family smoking exposure, chronic medical conditions, main motivation for quitting, and baseline vital signs showed no significant imbalance between the intervention and control groups. Overall, these findings support successful randomization and indicate that any subsequent between-group differences in cessation outcomes are unlikely to be attributable to baseline differences in participant characteristics.

### 3.2. Clinical Effectiveness Outcomes

Table 2 presents the primary and secondary cessation outcomes under the intention-to-treat analysis. The intervention demonstrated a clear and statistically significant benefit over usual care for the primary outcome of CO-verified continuous abstinence at 6 months. Specifically, 26.3% of participants in the intervention arm achieved continuous abstinence compared with 11.3% in the control arm, corresponding to nearly threefold higher adjusted odds of quitting in the intervention group (aOR = 2.84, 95% CI: 1.55–5.18; *p* < 0.001).

A consistent pattern was observed across all secondary abstinence outcomes. At 3 months, the 7-day point prevalence abstinence rate was 33.1% in the intervention group versus 15.6% in the control group (aOR = 2.68, 95% CI: 1.56–4.60; *p* < 0.001). This advantage persisted at 6 months, with higher rates of both 7-day point prevalence abstinence (30.6% vs. 14.4%; aOR = 2.62, 95% CI: 1.48–4.62; *p* = 0.001) and 30-day point prevalence abstinence (28.1% vs. 11.9%; aOR = 2.89, 95% CI: 1.59–5.24; *p* < 0.001) in the intervention arm.

Among baseline dual users, who represented a clinically challenging subgroup, the intervention also produced superior outcomes. Both-product continuous abstinence was achieved by 21.9% of dual users in the intervention arm compared with 7.8% in the control arm, with significantly higher adjusted odds of success in the intervention group (aOR = 3.30, 95% CI: 1.12–9.75; *p* = 0.026). In addition, partial cessation, defined as quitting one tobacco product while continuing the other, was observed in 12.5% of intervention participants and 9.4% of control participants. Among the 14 dual users who achieved partial cessation (8 intervention, 6 control), the majority quit waterpipe while continuing combustible cigarette smoking. While the intervention effect size was strong among exclusive waterpipe users (OR 3.30), this subgroup analysis did not reach statistical significance (*p* = 0.082), likely due to the small sample size.

### 3.3. Pharmacotherapy Utilization

Table 3 shows that the intervention was associated with a substantial increase in the initiation and maintenance of evidence-based smoking cessation pharmacotherapy. Overall, 72.5% of participants in the intervention group initiated pharmacotherapy, compared with only 28.1% in the control group, showing a highly significant difference (*p* < 0.001) between the groups. Moreover, in dual users, the intervention was associated with the intensive use of NRT compared with the control group (62.5% vs. 3.1%, *p* < 0.001).

Regarding adherence, the results showed that in participants who initiated pharmacotherapy, the percentage of participants with continuous self-reported use of the medication for at least 8 weeks was higher in the intervention group (56.0%) than in the control group (26.7%) (*p* = 0.002).

### 3.4. Digital Engagement (Intervention Arm Only)

The engagement with the digital support layer by participants within the intervention arm is presented in Table 4. The participant engagement with the WhatsApp^®^-based pathway for the follow-up remained high across the intervention period. Specifically, 88.1% responded to the early support contact within Week 1, 73.8% completed the contact for consolidation within Week 4, and 61.3% remained digitally engaged with the intervention for the Month 6 contact. Across the 6-month intervention, the automated system detected 145 red flag triggers from 62 unique participants. These triggers included situations such as severe cravings, lapse, or other relapse risks. Critically, 84.1% of these escalations received a successful telepharmacy call with a pharmacist within 48 h.

### 3.5. Implementation Outcomes

In accordance with the Hybrid Type 2 effectiveness-implementation design, the operational feasibility of the Dual-Quit Digital model was evaluated. At the cluster level, service adoption and retention were absolute, with all 8 randomized intervention pharmacies remaining active throughout the 6-month follow-up without withdrawal. Patient-level reach and recruitment feasibility were similarly successful, smoothly achieving the predefined enrollment target of 160 intervention participants (Section 3.1).

Furthermore, the implementation of the phenotype-stratified consultation workflow successfully drove clinical action, resulting in significantly higher rates of evidence-based pharmacotherapy initiation and longitudinal adherence compared to usual care (Table 3). Finally, the offloaded digital architecture proved highly feasible in practice. The high response rates to automated touchpoints and the 84.1% success rate of pharmacist telepharmacy escalations (Table 4) indicate that the continuous follow-up layer can be seamlessly integrated into real-world pharmacy operations without overwhelming standard counter workflows.

## 4. Discussion

The present study evaluated the effectiveness and real-world implementation of the Dual-Quit Digital pharmacy service—a novel, culturally tailored cessation program (i.e., specifically addressing waterpipe use which is deeply culturally embedded in the region, focusing on relevant family and social smoking triggers, and utilizing WhatsApp^®^ which is the overwhelmingly preferred communication platform in the Middle East) for combustible and dual-tobacco users in Jordan. Utilizing a pragmatic, Hybrid Type 2 cluster-randomized controlled trial design, the study aimed to address critical gaps in traditional face-to-face pharmacy models, such as high patient attrition and a lack of protocols for complex tobacco use behaviors.

Males were dominant in this trial which is representative of the combustible tobacco-using population seeking care in Jordan, where male smoking prevalence vastly outweighs female prevalence [4]. This cluster-randomized trial found that the Dual-Quit Digital intervention produced clinically and statistically significant improvements in smoking-cessation outcomes compared with usual care. Participants in the intervention arm had higher CO-verified continuous abstinence at 6 months, as well as higher 7-day point prevalence abstinence at 3 and 6 months and higher 30-day point prevalence abstinence at 6 months. Importantly, the intervention also improved both-product abstinence among baseline dual users, a subgroup that is often underrepresented in cessation trials. These clinical gains were accompanied by markedly higher uptake of evidence-based pharmacotherapy, better self-reported adherence to pharmacotherapy, and sustained engagement with the WhatsApp^®^ follow-up layer, suggesting that the intervention’s effect was driven not by a single component but by the combination of structured pharmacist counselling, phenotype-stratified pharmacotherapy support, and longitudinal digital relapse-prevention. This interpretation is consistent with broader cessation evidence showing that behavioral counselling and pharmacotherapy are each effective, but are most effective when combined, and with community-pharmacy evidence indicating that more intensive pharmacist-delivered support can improve quit outcomes relative to less intensive care [3,5].

The magnitude of effect observed in the present trial is broadly consistent with prior evidence supporting pharmacist-led cessation care, while also suggesting that a more structured and digitally supported model may yield stronger outcomes in some settings. Carson-Chahhoud et al. [5] found that intensive pharmacy-personnel interventions approximately doubled long-term quit rates compared with less intensive support, and Phillips et al. [16] reported 6-month quit rates of 22% to 36% across pharmacist-led smoking-cessation programs in Canada. Our 6-month continuous abstinence rate in the intervention arm falls within that range, which supports the plausibility of the observed effect. At the same time, our results appear stronger than those reported in the Qatar pharmacist-led trial [15], where cessation rates favored the intervention but did not reach statistical significance. A likely explanation is that the present model moved beyond training and face-to-face counselling alone by embedding a standardized workflow, phenotype-based treatment decisions, and ongoing digital support over 6 months. This may also explain why our findings contrast with the STOP trial, in which pharmacy-staff training improved perceived skills but did not significantly increase quit-service throughput, retention, or quit rates; in our study, training was paired with a concrete operational pathway and an escalation mechanism rather than functioning as a stand-alone implementation strategy [5,8,15,16].

The digital component is another plausible contributor to the intervention’s effectiveness. Evidence from the Cochrane review by Whittaker et al. [9] indicates that automated text-messaging interventions improve quit rates compared with minimal support and can add benefit when layered onto other cessation support. Our findings are aligned with that literature. Although active response rates to the WhatsApp^®^ component decreased from 88.1% to 61.3% over six months, this represents robust, sustained engagement when compared to typical mobile health (mHealth) interventions, which often experience steep attrition dropping below 20–30% within just a few weeks. Furthermore, most red-flag escalations were followed by pharmacist contact within 48 h, indicating that the WhatsApp^®^ pathway functioned not merely as a reminder tool but as an active relapse-prevention channel. This differs from the PharmQuit trial reported by Asayut et al. [23], in which adding a smartphone app to pharmacist counselling did not significantly improve abstinence over counselling alone. One possible reason for the difference is that the digital layer in our study was integrated into pharmacist workflow, used simple and familiar messaging technology, and triggered human follow-up when risk signals emerged, rather than relying primarily on passive app engagement.

The dual-user findings are particularly important in view of the limited evidence base for waterpipe and dual-tobacco cessation. Maziak et al. [7] concluded that behavioral interventions can improve waterpipe cessation, but also highlighted the scarcity of robust evidence for pharmacological approaches and the lack of targeted studies for dual or polytobacco users. Our subgroup results therefore add useful evidence by showing that a phenotype-stratified community-pharmacy model can improve both-product abstinence among people using cigarettes and waterpipe concurrently. This is especially relevant in Jordan and the wider Eastern Mediterranean region, where waterpipe use is culturally embedded and where pharmacists are highly accessible but historically underused in cessation care. In Northern Jordan, Sakka et al. [6] found that pharmacists generally had positive attitudes toward smoking-cessation services, yet reported low practice levels because of limited training, the absence of structured cessation programs, and low patient demand. The present findings suggest that these barriers may be at least partly modifiable when pharmacists are supported with standardized workflows, documentation tools, and a practical follow-up system rather than being expected to provide cessation support opportunistically [6,7].

This study has several strengths. First, the cluster-randomized design reduced contamination across pharmacy staff within the same site and enhanced real-world relevance. Second, the use of ITT analysis provides a conservative estimate of intervention effectiveness. Third, abstinence was biochemically verified using exhaled carbon monoxide at the primary endpoint, which strengthens internal validity compared with self-report alone and is consistent with recommendations for smoking-cessation trials when feasible [21]. Fourth, the intervention addressed an important evidence gap by explicitly targeting dual users rather than assuming that cigarette-focused algorithms are sufficient for all tobacco phenotypes. Additionally, because patient participants were only consented for the care model available at their specific pharmacy, they were blinded to the overall trial design, effectively reducing expectation or disappointment bias. Finally, the inclusion of implementation-relevant outcomes, such as cluster retention, digital engagement, and escalation responsiveness, adds practical value by showing not only that the intervention worked, but also that it could be delivered within routine community-pharmacy practice.

Several limitations should also be acknowledged. First, while the intervention more than doubled the quit rate compared to usual care, it must be noted that nearly three-quarters (73.7%) of the intervention group did not achieve continuous abstinence, reflecting the well-documented ceiling effects and limitations of traditional NRT-based cessation models. Second, the study was not powered to stratify and compare success rates between light and heavy smokers. Third, while exhaled CO effectively validates combustible tobacco abstinence, cotinine/saliva tests were deliberately excluded due to high rates of NRT usage. Fourth, although the primary clinical endpoint was biochemically verified, some secondary measures, including pharmacotherapy adherence and parts of the digital-engagement pathway, relied on self-report or routine service documentation and may therefore be subject to reporting bias. Consequently, the study’s biochemical verification possesses limited specificity regarding the potential substitution of non-combustible electronic nicotine delivery systems. Moreover, the intervention necessitated smartphone ownership and active WhatsApp^®^ access, which, while highly prevalent in Jordan, could theoretically exclude populations lacking digital literacy or smartphone access. Finally, the primary clinical endpoint was assessed at 6 months; longer-term follow-up (e.g., 12 months) is required to determine whether abstinence is sustained after the digital maintenance and relapse-prevention pathway officially concludes. Overall, however, the findings support the Dual-Quit Digital model as a pragmatic and scalable approach for improving smoking cessation outcomes in a setting where structured pharmacist-led tobacco treatment has been needed but insufficiently operationalized.

## 5. Conclusions

This pragmatic cluster randomized controlled trial demonstrated that the Dual-Quit Digital model significantly improved 6-month CO-verified continuous abstinence compared with usual care. The intervention also enhanced secondary abstinence outcomes, benefited baseline dual users, and increased evidence-based pharmacotherapy initiation and adherence. High participant engagement and successful telepharmacy escalations confirmed its real-world operational viability. Overall, integrating phenotype-stratified pharmacist counselling, pharmacotherapy support, and low-burden digital follow-up offers a pragmatic cessation model for settings with prevalent cigarette and waterpipe co-use. Future research should investigate longer-term abstinence, broader implementation, cost-effectiveness, and harm-reduction alternatives for patients unable to quit via standard care.

## Figures and Tables

**Table 1:** Demographic and tobacco use variables compared between the two study groups.

Variable	Intervention (N = 160)	Control (N = 160)	*p*-Value *
**Age in years, n (%)**			0.612
18–29	38 (23.8%)	42 (26.3%)	
30–39	62 (38.8%)	58 (36.3%)	
40–49	36 (22.5%)	35 (21.9%)	
≥50	24 (15.0%)	25 (15.6%)	
**Gender (males), n (%)**	148 (92.5%)	151 (94.4%)	0.485
**Highest educational level, n (%)**			0.741
Primary	15 (9.4%)	18 (11.3%)	
Secondary/high school	45 (28.1%)	40 (25.0%)	
College diploma	35 (21.9%)	42 (26.3%)	
Undergraduate degree	50 (31.3%)	45 (28.1%)	
Postgraduate degree	15 (9.4%)	15 (9.4%)	
**Marital Status, n (%)**			0.598
Single	38 (23.8%)	42 (26.3%)	
Married	76 (47.5%)	71 (44.4%)	
Others	46 (28.7%)	47 (29.3%)	
**Baseline Tobacco-Use Phenotype, n (%)**			0.884
Exclusive cigarette smoker	72 (45.0%)	70 (43.8%)	
Exclusive waterpipe/shisha user	24 (15.0%)	26 (16.3%)	
Dual user (cigarette + waterpipe)	64 (40.0%)	64 (40.0%)	
**Years of Tobacco-Use, n (%)**			0.814
0 to 4.99 years	16 (10.0%)	15 (9.4%)	
5 to 10 years	26 (16.3%)	23 (14.4%)	
More than 10 years	118 (73.8%)	122 (76.3%)	
**Number of cigarettes per day (Mean ± SD)** (n = 136 vs. 134) †	18.4 ± 8.2	19.1 ± 9.0	0.495
**Waterpipe sessions per week (Mean ± SD)** ‡ (n = 88 vs. 90) †	3.2 ± 2.1	3.5 ± 2.4	0.380
**Baseline FTND score (Mean ± SD)**	5.4 ± 2.1	5.1 ± 2.3	0.224
**Baseline exhaled CO (ppm) (Mean ± SD)**	17.2 ± 6.4	16.8 ± 7.1	0.591
**Tried quitting before (Yes), n (%)**	110 (68.8%)	105 (65.6%)	0.540
**Family member smokes (Yes), n (%)**	98 (61.3%)	102 (63.8%)	0.638
**Presence of chronic medical conditions, n (%)**	40 (25.0%)	38 (23.8%)	0.795
Hypertension	18 (11.3%)	15 (9.4%)	0.573
Diabetes	14 (8.8%)	12 (7.5%)	0.681
Asthma/chronic lung diseases	6 (3.8%)	8 (5.0%)	0.584
Cardiovascular diseases	4 (2.5%)	5 (3.1%)	0.735
Other conditions	2 (1.3%)	3 (1.9%)	0.655
**Most important reason for quitting, n (%)**			0.622
To live healthier	85 (53.1%)	78 (48.8%)	
Financial reasons	32 (20.0%)	36 (22.5%)	
Religious reasons	20 (12.5%)	24 (15.0%)	
To be a role model for children	18 (11.3%)	16 (10.0%)	
Pressure from family/healthcare providers	5 (3.1%)	6 (3.8%)	
**Baseline Vitals (Mean ± SD)**			
Heart rate (bpm)	78.4 ± 12.1	79.1 ± 11.8	0.590
Systolic blood pressure (mmHg)	124.5 ± 13.2	125.8 ± 14.1	0.388
Diastolic blood pressure (mmHg)	78.2 ± 8.5	79.0 ± 9.1	0.412
Body Mass Index (BMI)	28.4 ± 5.2	27.9 ± 4.8	0.370

* *p*-values account for cluster-level variance. Evaluated using chi-squared tests for categorical variables and independent t-tests for continuous variables. † Calculated among exclusive cigarette and dual users only. ‡ Calculated among exclusive waterpipe and dual users only. FTND = Fagerström Test for Nicotine Dependence; CO = Carbon Monoxide.

**Table 2:** Primary and Secondary Cessation Outcomes (ITT Analysis, N = 320).

Outcome Measure	Intervention (n = 160)	Control (n = 160)	Adjusted OR (95% CI) *	*p*-Value
**Primary Outcome**				
Continuous abstinence at 6 months (CO-verified)	42 (26.3%)	18 (11.3%)	2.84 (1.55–5.18)	<0.001
**Secondary Outcomes**				
7-day PPA at 3 months	53 (33.1%)	25 (15.6%)	2.68 (1.56–4.60)	<0.001
7-day PPA at 6 months	49 (30.6%)	23 (14.4%)	2.62 (1.48–4.62)	0.001
30-day PPA at 6 months	45 (28.1%)	19 (11.9%)	2.89 (1.59–5.24)	<0.001
**Subgroup: Dual-Users (n = 64 per arm)**				
Both-product continuous abstinence	14 (21.9%)	5 (7.8%)	3.30 (1.12–9.75)	0.026
Partial cessation (*quit one*, *continued other*) †	8 (12.5%)	6 (9.4%)	-	-
**Subgroup: Exclusive Cigarette (n = 72 Intervention, n = 70 Control)**				
Continuous abstinence	19 (26.4%)	9 (12.9%)	2.43 (1.03–5.75)	0.042
Subgroup: Exclusive Waterpipe (n = 24 Intervention, n = 26 Control)				
Continuous abstinence	9 (37.5%)	4 (15.4%)	3.30 (0.86–12.68)	0.082

* Adjusted for cluster-level variance using mixed-effects logistic regression. † Partial cessation is reported as a descriptive frequency only; no formal hypothesis testing or regression modeling was performed for this observational sub-category. (PPA = Point Prevalence Abstinence).

**Table 3:** Pharmacotherapy Utilization and Adherence.

Pharmacotherapy Measure	Intervention (n = 160)	Control (n = 160)	*p*-Value *
Initiated any evidence-based pharmacotherapy (e.g., NRT or prescription medication)	116/160 (72.5%)	45/160 (28.1%)	<0.001
Utilized NRT (Among dual-users)	40/64 (62.5%)	2/64 (3.1%)	<0.001
Adherence to Pharmacotherapy (Self-reported continuous use for ≥8 weeks among those who initiated)	65/116 (56.0%)	12/45 (26.7%)	0.002

* *p*-values account for cluster-level variance.

**Table 4:** Digital Engagement & Telepharmacy Escalation (Intervention Arm Only, N = 160).

Engagement Metric	Intervention Participants
**WhatsApp^®^ Touchpoint Response Rates**	
Responded to Touch 2 (Early digital support at Week 1)	141 (88.1%)
Completed Touch 3 (Consolidation support at Week 4)	118 (73.8%)
Completed Touch 4 (Maintenance support at Month 3)	108 (67.5%)
Remained digitally engaged up to Touch 5 (Month 6)	98 (61.3%)
**Automated System Escalations (6-Month Period)**	
Total “red-flag” triggers captured (e.g., severe cravings, lapses)	145 triggers
Unique participants who triggered a red-flag	62 participants
Successful pharmacist telepharmacy follow-up call within 48 h	84.1% of escalations

## Data Availability

Data are available as Supplementary Material file.

## Supplementary Materials

The following supporting information can be downloaded at: https://www.mdpi.com/article/10.3390/pharmacy14030077/s1.
